# PEDOT:Nafion for Highly Efficient Supercapacitors

**DOI:** 10.1021/acsami.4c01085

**Published:** 2024-04-23

**Authors:** Małgorzata Skorupa, Krzysztof Karoń, Edoardo Marchini, Stefano Caramori, Sandra Pluczyk-Małek, Katarzyna Krukiewicz, Stefano Carli

**Affiliations:** † Department of Physical Chemistry and Technology of Polymers, Silesian University of Technology, M. Strzody 9, Gliwice 44-100, Poland; ‡ Joint Doctoral School, Silesian University of Technology, Akademicka 2A, Gliwice 44-100, Poland; § Centre for Organic and Nanohybrid Electronics, Silesian University of Technology, S. Konarskiego 22B, Gliwice 44-100, Poland; ∥ Department of Chemical, Pharmaceutical and Agricultural Sciences, University of Ferrara, Ferrara 44121, Italy; ⊥ Department of Environmental and Prevention Sciences, University of Ferrara, Ferrara 44121, Italy

**Keywords:** capacitance, Nafion, PEDOT, secondary doping, supercapacitor

## Abstract

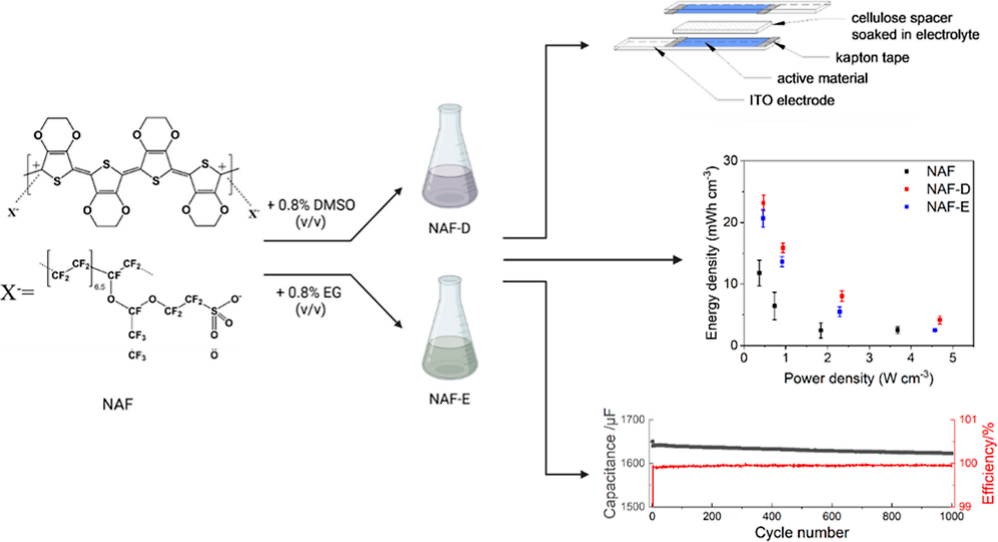

Supercapacitors offer notable properties as energy storage devices, providing high power density and fast charging and discharging while maintaining a long cycling lifetime. Although poly(3,4-ethylenedioxythiophene) doped with poly(4-styrenesulfonate) (PEDOT/PSS) has become a gold standard among organic electronics materials, researchers are still investigating ways to further improve its capacitive characteristics. In this work, we introduced Nafion as an alternative polymeric counterion to PSS to form highly capacitive PEDOT/Nafion; its advantageous supercapacitive properties were further improved by treatment with either dimethyl sulfoxide or ethylene glycol. Accordingly, electrochemical characterization of PEDOT/Nafion films revealed their high areal capacitance (22 mF cm^–2^ at 10 mV/s) and low charge transfer resistance (∼380 Ω), together with excellent volumetric capacitance (74 F cm^–3^), Coulombic efficiency (99%), and an energy density of 23.1 ± 1.5 mWh cm^–3^ at a power density of 0.5 W cm^–3^, resulting from a more effective ion diffusion inside the conductive film, as confirmed by the results of spectroscopic studies. A proof-of-concept symmetric supercapacitor based on PEDOT/Nafion was characterized with a specific capacitance of approximately 15.7 F g^–1^ and impressive long-term stability (Coulombic efficiency ∼99% and capacitance ∼98.7% after 1000 charging/discharging cycles), overperforming the device based on PEDOT/PSS.

## Introduction

The need for abandoning fossil fuels implies the diversification of energy generation sources and the rapid transfer into renewable energy sources. Although the sun and wind are excellent sources of clean energy, they are not easily dispatchable due to their intrinsically intermittent nature.^1,2^ To efficiently use renewable energy sources, it is required to develop efficient and inexpensive energy storage means. The design of efficient supercapacitors is, therefore, one of the most important research areas in today’s society.^3−6^ Supercapacitors, next to batteries and fuel cells, are energy storage devices endowed with attractive and promising properties, like high power density and unparalleled fast charging and discharging while maintaining long cycling lifetime.^3,7^ Depending on the charge storage mechanism, supercapacitors can be divided into two groups: electrical double layer capacitors based on electrostatic interactions and pseudocapacitors utilizing Faradaic reactions.^4^ In the latter group, the materials undergoing redox processes include metal oxides, conducting polymers (CPs), and hybrid materials.^8,9^ However, the use of metal oxides, when compared to CPs, is unfavorable due to their high costs, toxicity, low processability, and limited capacitance.^10^

CPs are the most commonly investigated pseudocapacitive materials, and they include polypyrrole (PPy), polyaniline (PANI), polythiophene (PTh), as well as their derivatives.^11^ Among the CPs, poly(3,4-ethylenedioxythiophene) doped with poly(4-styrenesulfonate) (PEDOT/PSS) is one of the most successful materials suitable for a variety of organic electronics applications thanks to its exceptional stability, commercial availability as a water dispersion, processability, and low price.^12,13^ In a pristine form, PEDOT/PSS exhibits low conductivity (from 0.1 to 1 S cm^–1^^14^); therefore, it is usually modified by different approaches, including various ways of synthesis and post-treatment methods.^14^ In particular, treatment with polar solvents like dimethyl sulfoxide (DMSO) or ethylene glycol (EG) has been indicated as an efficient way to largely increase the conductivity of PEDOT/PSS, reaching over 1200 S cm^–1^.^15,16^ This “secondary doping” can be achieved either by addition of the solvent directly to the PEDOT/PSS dispersion or as a post-treatment of the polymer film.^17^ The substantial increase in conductivity stems from the phase separation between conductive PEDOT and insulating PSS chains.^15,18^ In the case of EG treatment, the improved properties of PEDOT/PSS are explained by the increase in carrier mobility and density.^14,19^ A theoretical study on PEDOT/PSS enhanced with DMSO also showed that the main interaction appears between DMSO and neutral PSS and involves dissolution of the insulating PSS barrier.^20^

It is clear that even though the presence of PSS is beneficial from the point of view of polymer processing, since it allows PEDOT to be dispersed under aqueous conditions, its presence has a deteriorating effect on polymer’s conductivity. Consequently, looking for a more effective counterion for PEDOT is one of the challenges of organic electronics.^21^ Nafion is a copolymer in the form of a tetrafluoroethylene hydrophobic backbone containing highly hydrophilic sulfonate groups.^13^ As a water-soluble ionomer with high ionic conductance, Nafion has been commonly used in proton conducting membranes.^22^ Recently, it has been studied as a polyanionic dopant of PEDOT, as an alternative to PSS,^13,23−26^ resulting in the formation of PEDOT/Nafion, which was found to be easily dispersed in an aqueous medium.^25^ PEDOT/Nafion exhibits similar conductivity, but improved stability,^24^ capacitance,^26^ and adhesion to substrates^25^ when compared to PEDOT/PSS.^23^ Besides, Nafion is a well-known cation exchange polymer, exhibiting superselectivity and facile cation transport.^27^ These exceptional properties are based on the microstructure of Nafion, which consists of segregated domains of fluorocarbon and clusters of hydrated sulfonate sites, allowing the transport of cations through interconnected hydrated domains.

In this paper, we investigated the performance of PEDOT/Nafion films as potential pseudocapacitive materials. Coatings were obtained by depositing the water dispersion of PEDOT/Nafion on the electrodes and subjecting to either DMSO or EG treatment (hereafter referred as NAF, NAF-D, and NAF-E, respectively), assuming a similar secondary doping-based enhancement in electrochemical performance as previously noted for PEDOT/PSS.^14,15,17−20,28^ Thus, NAF, NAF-D, and NAF-E have been investigated with the use of electrochemical techniques to estimate their capacitance, rate capability, power density, energy density, and Coulombic efficiency. Structural and electrochemical characterizations were achieved by means of infrared, Raman, and UV/vis spectroscopies to corroborate results by electrochemical characterization. Microscopic investigation with the use of profilometry, scanning electron microscopy, and atomic force microscopy allowed us to assess the surface morphology of investigated materials, as well as their stability during electrochemical treatment. To verify the applicability of PEDOT/Nafion as an energy storage material, a symmetric supercapacitor was fabricated and its performance was examined through a repetitive constant current charging/discharging process.

## Materials and Methods

### Materials

All chemicals were purchased from Sigma-Aldrich except otherwise specified.

### Sample Preparation

**Figure 1:**
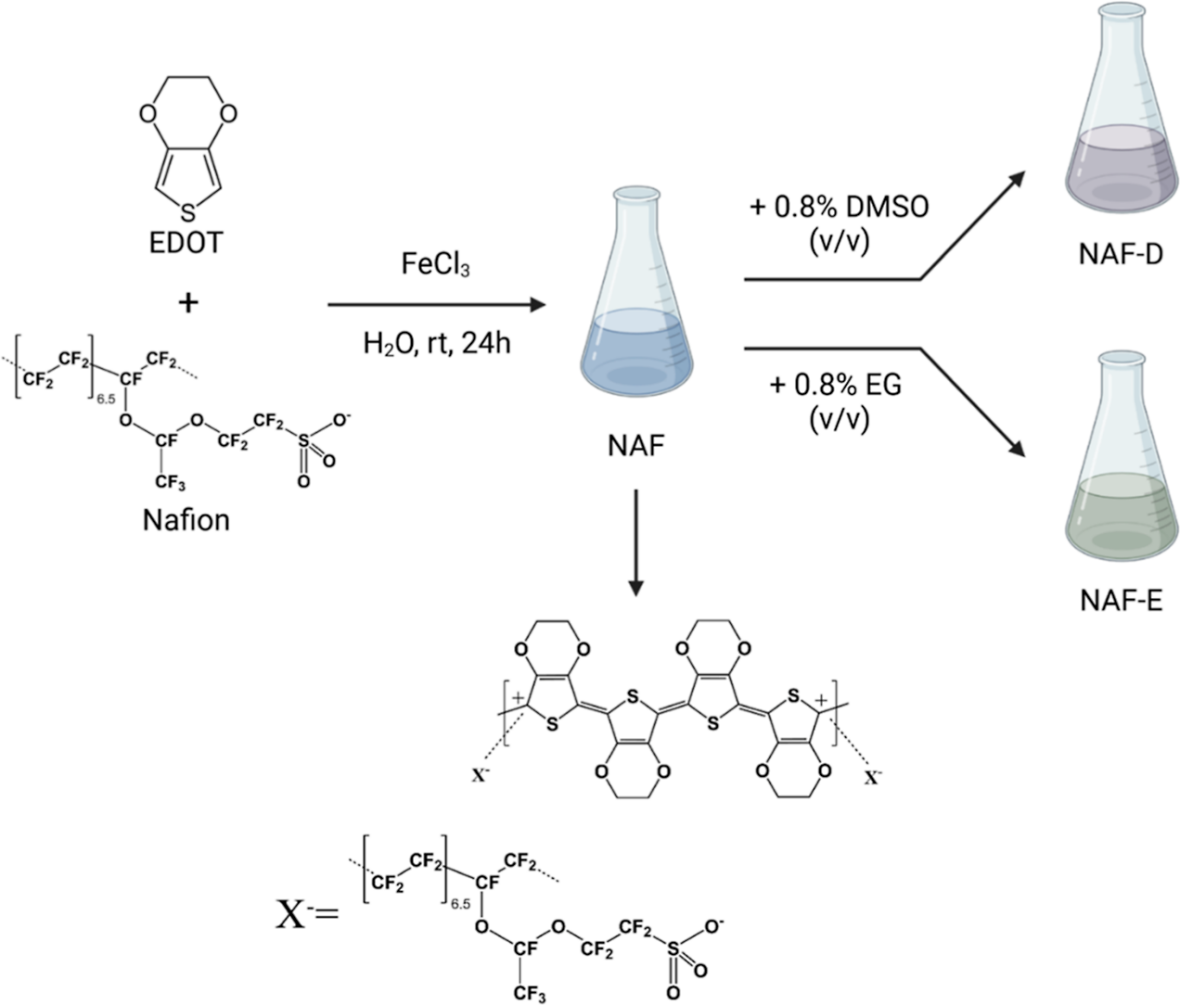
Schematic representation of the synthetic approach for PEDOT/Nafion (NAF) and NAF post-treated with dimethyl sulfoxide, DMSO (NAF-D), and ethylene glycol, EG (NAF-E).

The water dispersion of PEDOT/Nafion (NAF) (solid content of 4.14 ± 0.04%) was prepared and purified according to a published procedure (Figure 1).^25^ In short, it was sonicated at room temperature for 30 min to obtain a stable and homogeneous dispersion. As-prepared NAF dispersion was diluted to 1:6 v/v in milli-Q water, and in the cases of NAF-D and NAF-E, 0.8% v/v DMSO or EG was further added. Coatings of NAF, NAF-D, and NAF-E (NAFs) were prepared by drop-casting 180 μL of the water dispersion onto fluorine-doped tin oxide (FTO) electrodes (NSG) or glass slides. The deposition area (2.0 × 1.5) cm^2^ was delimited by applying a Surlyn 25 mask.

### Spectroscopic Analysis

UV–vis spectra of NAF, NAF-D, and NAF-E films coated on glass slides were recorded in the 400–800 nm spectral range with a Cary 300 UV–vis Spectrophotometer (Agilent Technologies). Attenuated total reflection infrared spectroscopy (ATR–FTIR) spectra were recorded with the use of an IR PerkinElmer Spectrum Two with UATR diamond accessory, in the spectral range between 450 and 3000 cm^–1^ and a spectral resolution of 2 cm^–1^. Raman spectra for NAF, NAF-D, and NAF-E films deposited on glass slides were acquired using an Edinburgh FS920 spectrofluorimeter equipped with a 189 mW CW 532 nm laser as the excitation source and a photomultiplier tube as the detector. The emission slit was set at 0.1 nm, 300 scans sampled at a 0.1 nm step were averaged to achieve an acceptable S/N ratio. Raman spectra of doped and dedoped forms of PEDOT/Nafion film were collected before and after chemical reduction. In order to achieve chemical reduction (dedoping), the coated film was treated with a hydrazine solution, according to a published paper.^29^ Raman peaks were fit by using a Gaussian profile.

### Surface Characterization

Morphological characterization was performed using an optical profilometer (Filmetrics Profilm 150 3D, KLA Co.), a scanning electron microscope (Phenom Pro X) equipped with 3D roughness reconstruction software operating at an accelerating voltage of 15 kV, and an atomic force microscope (CoreAFM Nanosurf) with the application of a phase contrast (tapping mode) using Tapping Mode HQ/NSC15/AlBS AFM Probe (MikroMasch). Roughness was calculated with the use of AFM data as a surface roughness (S_a_). Gwyddion SPM software was applied to process the AFM images and to determine the surface roughness.

### Electrochemical Analysis

Voltammetric experiments were carried out by means of a CHI 660c electrochemical workstation in a three-electrode system, comprising Ag/AgCl (3 M KCl) reference electrode, Pt foil (1 cm^2^) auxiliary electrode, and a NAFs-coated FTO working electrode. Cyclic voltammetric (CV) curves were collected in 1 M Na_2_SO_4_ solution, in the potential range from −0.5 to 1.0 V (vs Ag/AgCl) at scan rates of 10, 20, 50, 100, and 200 mV s^–1^. CV curves were then used to determine the integrated areal capacitance (C_CV(A)_, F cm^–2^) according to the formula^30^
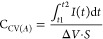
1where *t*_1_ denotes the beginning of a CV cycle, *t*_2_ denotes the end of a CV cycle, *I* denotes current (*A*), Δ*V* denotes potential range (*V*), and *S* denotes geometrical area of a polymer-coated electrode (cm^2^).

FTO electrodes coated with NAFs were also analyzed in the presence of a reversible redox probe, potassium hexacyanoferrate (III). CV scans were collected in 0.1 M KCl solution containing 5 mM K_3_[Fe(CN)_6_] within a potential range from 0.6 V to −0.1 V (vs Ag/AgCl) at a scan rate of 5 mV s^–1^. Electroactive surface area *A* (cm^2^) was calculated according to the formula^31^
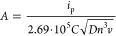
2where *i*_p_ is the reduction/oxidation peak current (*A*), *n* is the number of electrons contributing to the redox reaction, *C* is the concentration of Fe(CN)_6_^3–^ in the bulk solution (mol cm^–3^), *D* is the diffusion coefficient of Fe(CN)_6_^3–^ in KCl solution, and *v* is the scan rate (V s^–1^).

The impedance measurements were carried out in 0.1 M KCl solution in a frequency range from 100 kHz to 100 mHz, at an AC voltage amplitude of 50 mV (vs Ag/AgCl) and a DC potential of 0 V (vs Ag/AgCl). EIS Spectrum Analyzer 1.0 software^32^ and the Powell algorithm were used to fit the experimental data to an equivalent electrical circuit model.

Electrochemical charging/discharging processes were performed by means of a galvanostatic mode in 1 M Na_2_SO_4_ solution due to its common use in the characterization of supercapacitors allowing for comparing the collected results with previous literature studies.^33,34^ FTO electrodes coated with NAFs were subjected to the current densities of 0.1, 0.2, 0.5, and 1 mA cm^–2^ until the potential of 1.0 V (vs Ag/AgCl) was reached. The process of discharging was monitored until the potential of −0.5 V (vs Ag/AgCl) was reached. Areal capacitance (C_GCD(A)_, F cm^–2^) was calculated according to the following formula^35^
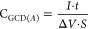
3where *I* denotes discharging current (*A*), *t* denotes discharging time (s), Δ*V* denotes discharging potential difference (*V*), and *S* denotes geometrical area of an electrode (cm^2^).

Volumetric capacitance (C_GCD(V)_, F cm^–3^) was calculated by dividing C_GCD(A)_ by the thickness of a polymer film (cm).

Specific capacitance (C_GCD(S)_, F g^–1^) was calculated according to the following formula
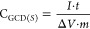
4where *I* denotes discharging current (*A*), *t* denotes discharging time (*s*), Δ*V* denotes discharging potential difference (*V*), and *m* is the mass of the polymer film (*g*).

Coulombic efficiency (η,%) was calculated according to the following formula^36^
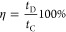
5where *t*_D_ and *t*_C_ are the discharging time (*s*) and the charging time (*s*), respectively.

Energy density (E, mWh cm^–3^) was calculated according to the following formula^36^
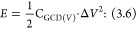
6where C_GCD(*V*)_ is the volumetric capacitance (F cm^–3^) and Δ*V* denotes the discharging potential difference (*V*).

Power density (P, W cm^–3^) was calculated according to the following formula^36^
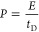
7where *E* is the energy density (mWh cm^–2^).

The measurements were performed in triplicate, and the results were expressed as a mean ± standard deviation.

### Supercapacitors Devices Preparation and Testing

To prove the ability of PEDOT/Nafion to serve as a good capacitive material, various symmetric supercapacitors were made where each electrode was covered with the same material (either PEDOT/PSS or NAF-D). ITO-covered glass electrodes (PGO, *R*_sq_ ≤ 20 Ω/□) were used as a support and current collector. The surface of each of them was limited to 2.25 cm^2^ by Kapton tape. Polymers were deposited by drop-casting, followed by heating on a hot plate. 180 μL of solution made by mixing 100 μL of aqueous dispersion of an active material (PEDOT/PSS or NAF-D, respectively), 5 μL of DMSO and 500 μL of deionized water, was applied uniformly on each electrode, and then, the electrodes were kept at 60 °C until solvent evaporation. Afterward, electrodes were transferred to a hot plate at 100 °C for 20 min. Each layer had 0.5 mg of active material.

SC devices were assembled immediately afterward. To avoid a short circuit, a separator made of a cellulose filter (thickness of 73 μm) was placed between electrodes. The filter separator was soaked in the electrolyte solution (0.2 M tetrabutylammonium hexafluorophosphate in a mixture of acetonitrile/propylene carbonate, 1:3 by volume). Fabricated SCs were tested with cyclic voltammetry and chronopotentiometry methods in a two-electrode system, i.e., with the reference and counter electrodes shorted. Initially, in CV experiments, the scan rate of 100 mV s^–1^ was applied and potential range was varied from 0 V – 1 V to 0 V – 2 V (vs Ag/AgCl). Later, the devices were examined with a potential range limited to 0 V – 1 V (vs Ag/AgCl), while the scan rate varied between 20 and 500 mV s^–1^. Chronopotentiometry was applied to examine capacitive characteristics by repetitive charging/discharging cycles with current in the range of 50–5000 μA, with limiting voltage set to 0 and 1 V for discharging and charging processes, respectively.

To study the pseudocapacitive contribution to electrochemical energy storage in NAF-based SC, a power law relationship was examined^37^
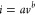
8where *i* is the current (*A*), *v* is the scan rate (V s^–1^), *a* is the adjustable parameter, and *b* is the exponent calculated from the plot ln (*i*) versus ln (*v*).

Data were analyzed for different potentials (0.4, 0.6, and 0.8 V).

## Results and Discussion

### Electrochemical Characterization

**Figure 2:**
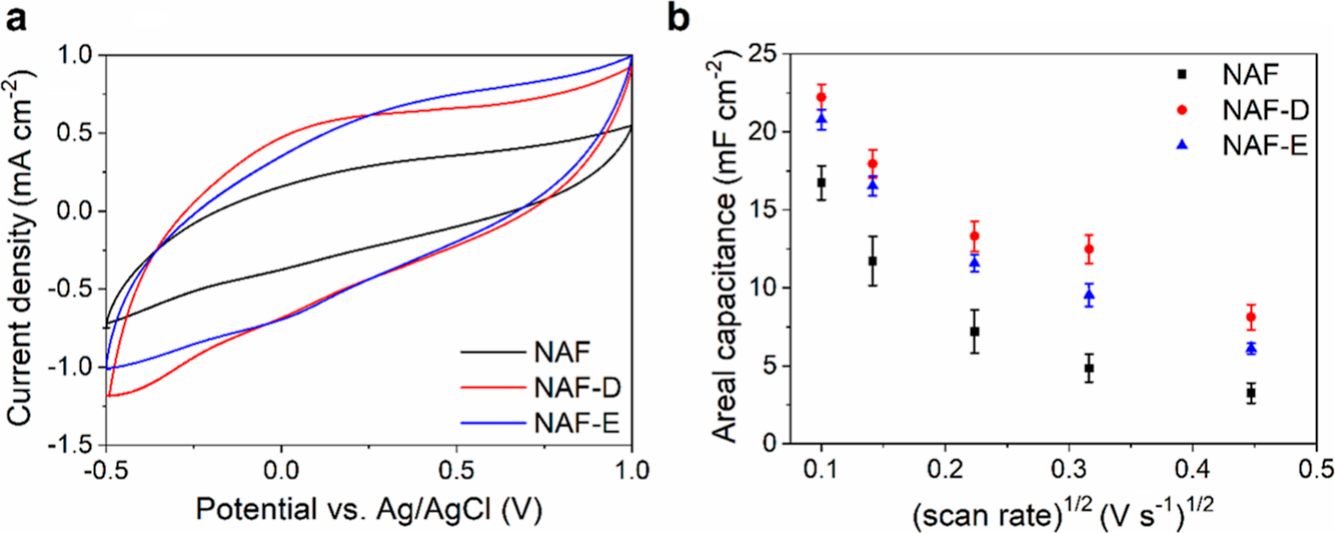
Electrochemical analysis of NAFs: (a) cyclic voltammetry curves collected in 1 M Na_2_SO_4_ at 100 mV s^–1^; (b) the effect of the scan rate on an areal capacitance of NAFs.

CV curves of NAF, NAF-D, and NAF-E revealed a typical capacitive behavior of PEDOT-based materials, as depicted in Figure 2a.^38−40^ The larger areas enclosed by the cyclic voltammograms that can be observed for both NAF-D and NAF-E are consistent with higher areal capacitances when compared to NAF, which is confirmed by the calculation of areal capacitance values according to eq 1 (Figure 2b). As the rate of electron transfer is related to the scan rate,^41^ the change in the scan rate is found to affect the capacitance of NAF, NAF-D, and NAF-E. The increase in the capacitance with a decrease in the scan rate, which is typical for supercapacitors, should be associated with the presence of inner or remote active sites characterized by sluggish electron transfer that require longer times for charge extraction to occur, thus being unable to follow the redox transitions completely at the highest scan rates.^42^ Therefore, the lowest scan rates, allowing more time for charge migration and collection, enable a more reliable estimation of the full capacitive character of an electrode material.^43^ NAF-D, in particular, is found to outperform both NAF and NAF-E, with the areal capacitance reaching 22.2 ± 0.8 mF cm^–2^ at a scan rate of 10 mV s^–1^. Other literature proceedings report the areal capacitance of pristine PEDOT/PSS between 4 mF cm^–2^^44^ and 10 mF cm^–2^,^45^ evidencing the efficiency of Nafion doping and DMSO treatment in the increase of the capacitive character of PEDOT, which could be partially related to the increase of its electroactive surface area (Figure S1).

It should be noted that, in general, the capacitance of PEDOT is strictly related to the number of charge carriers that are formed during the synthesis of the conductive polymer, but it should also be remembered that PEDOT is both an ohmic and an ionic conductor.^46^ Thus, the overall capacitance is strongly affected by the dynamics that control the access of the electrolyte to the innermost active sites of the conductive films.^46^ This translates into a larger capacitance of the film.

**Figure 3:**
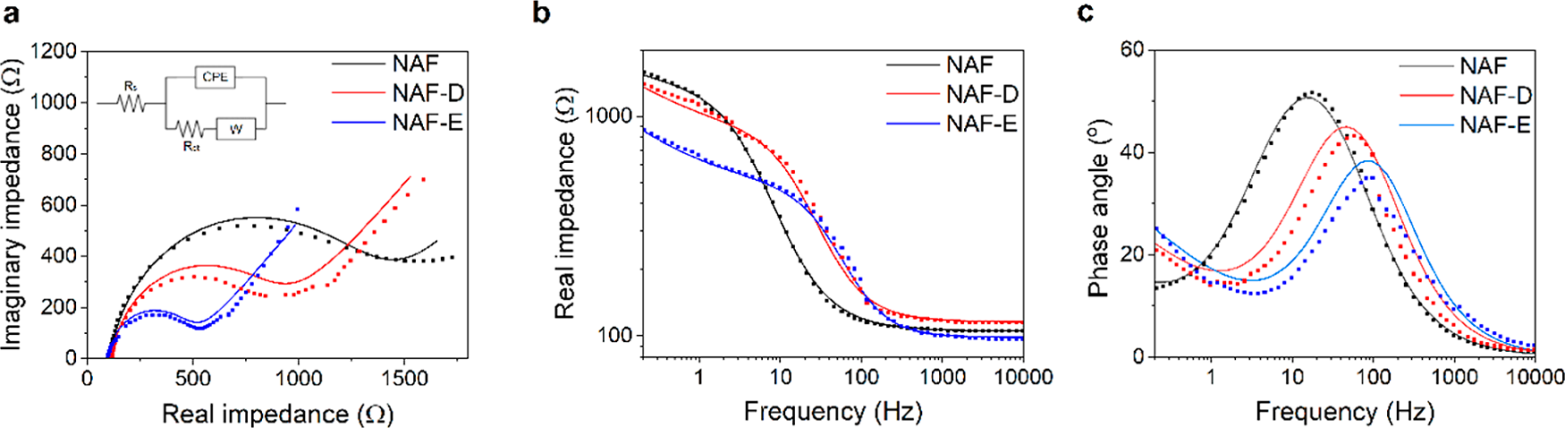
EIS spectra in the form of Nyquist and Bode plots of NAFs: (a) imaginary impedance vs real impedance (Nyquist plot) with an equivalent electric circuit as an inset; (b) real impedance vs frequency; and (c) phase angle vs frequency.

EIS spectra in the form of Nyquist and Bode plots (Figure 3) indicate a high similarity among the electrical properties of NAF, NAF-D, and NAF-E, based on essentially the same mechanism of charge transfer. In particular, in the midfrequency domain, the Nyquist plots are dominated by a semicircle (Figure 3a) with a time constant which ranges between 100 and 10 Hz. It was reported that the presence of this signal can be ascribed to the ion compensated charge transfer along the conductive polymer.^24^ In order to extract quantitative parameters, experimental EIS data were fitted according to an equivalent circuit model based on the solution resistance (*R*_S_) in series with a parallel element composed by a charge transfer resistance (*R*_CT_) and a double layer capacitance (C) to account for the charge transport across the conductive film. In addition, a Warburg element (W) was inserted in series with *R*_CT_ to describe the ionic contribution to the overall impedance, as depicted by the inset in Figure 3a. The magnitude of the Warburg impedance *Z*_W_ can be expressed by the following equation
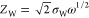
9where ω is the angular frequency (ω = 2π*f*, where *f* is the frequency in Hz) and σ_W_ is the Warburg coefficient.^31^

To improve the quality of the fitting, a constant phase element (CPE) was used instead of a pure capacitance. The impedance of the CPE (Z_CPE_) can be calculated according to equation
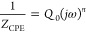
10where *j* is the imaginary unit and *Q*_0_ and *n* are the constant parameters of the CPE. The physical meaning of *Q*_0_ can be related to a pure capacitor when ω *=* 1 rad s^–1^^31^

As summarized in Table S1, the treatment of NAF with either DMSO or EG produces a reduction of the charge transfer resistance *R*_CT_, which is consistent with a faster hole transport, as outlined also by the increase of the characteristic frequency , and shown in Figure 3b–c.

### Spectroscopic Characterization

ATR–FTIR spectra of NAF, NAF-D, and NAF-E (Figure S2) are consistent with previous literature reports,^25,47^ with the bands assigned to PEDOT (1530 and 1310 cm^–1^ for C–C and C=C stretching modes for thiophene ring, 969 cm^–1^ for stretching mode in the C–O–C bond of ethylenedioxy residues, and 836 and 688 cm^–1^ for C–S bond vibration of the thiophene ring) and Nafion (1138 cm^–1^ for CF_2_ asymmetric stretching mode, 1193 cm^–1^ for asymmetric stretching of CF_2_ and SO_3_H, and 1051 cm^–1^ for symmetric stretching of the sulfonic groups). Additionally, ATR–FTIR spectrum of NAF-D contained signals associated with DMSO, namely, 1695 cm^–1^ for C=S bond stretching mode of the sulfoxide groups, as well as 2915 and 2850 cm^–1^ for symmetric and asymmetric stretching mode of CH_3_ groups in a sulfoxide ion.^48,49^ As previously reported,^50^ ATR–FTIR spectrum of NAF-E did not show any additional signals coming from the presence of EG, which could indicate that it is partially removed from NAF-E when in contact with water.

**Figure 4:**
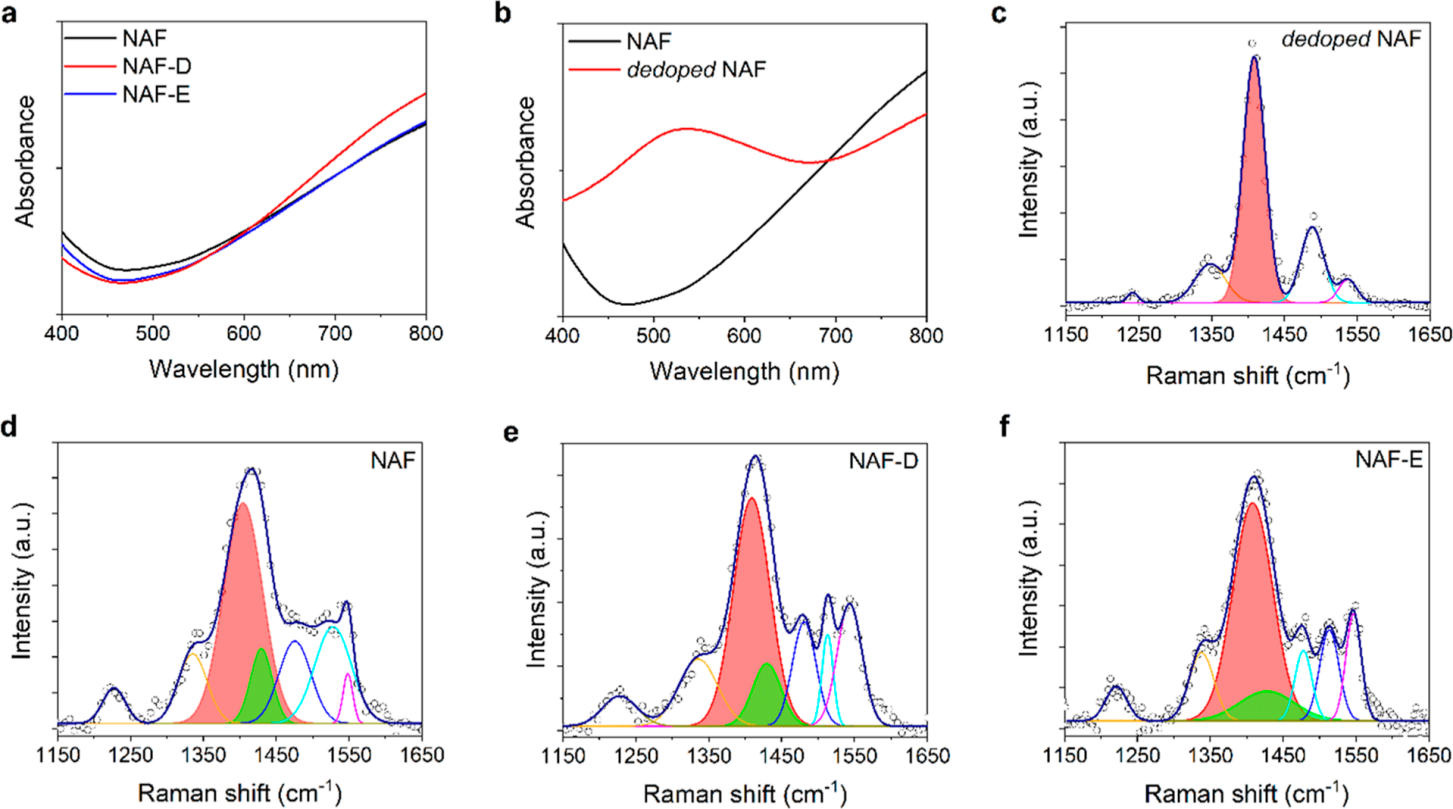
Characterization of NAF-based films: absorption spectra of (a) doped NAF, NAF-D, and NAF-E; (b) comparison between spectra of doped and dedoped NAF; and Raman spectra of (c) dedoped NAF, (d) NAF, (e) NAF-D, and (f) NAF-E (experimental and calculated data are reported as circles and lines, respectively): red and green areas are referred to the dedoped and doped PEDOT, respectively.

The typical absorption spectra of the NAFs films are reported in Figure 4a. The progressive increase in absorbance from 400 to 800 nm is due to the presence of polaron and bipolaron charge carriers in all investigated films, thereby confirming the oxidized and highly doped state of NAF, NAF-D, and NAF-E.^51^ The treatment of NAF with a reducing agent (hydrazine) leads to the appearance of a broad band at ∼520 nm, as displayed in Figure 4b. This new signal is consistent with the formation of a neutral or dedoped state of PEDOT.^52^

Raman spectroscopy has been used in the literature to estimate the doping level of PEDOT films.^53^ Raman spectrum of *neutral* (reduced) NAF (Figure 4c) is dominated by a strong signal at 1408 cm^–1^, which is linked to the C_α_ = C_β_ symmetric stretching vibration (Figure S3). The other less intense peaks are referred to the C_α_ = C_β_ asymmetric mode (1488 and 1536 cm^–1^), the C_β_-C_β_ deformation (1348 cm^–1^), and the C_α_-C_α’_ (1227 cm^–1^) inter-ring stretching vibration.^54^ In the cases of doped (oxidized) films (Figure 4d–f), a broadening, together with a slight red shift of the main signal at 1427 cm^–1^ can be observed, if compared to the Raman spectrum of *neutral* NAF. It comes from the fact that the C_α_ = C_β_ symmetric stretching band arises from the contribution of both the neutral and oxidized forms of PEDOT, with the latter’s signal centered at larger wavenumbers. It was reported that greater oxidation levels are expected for larger shifts of the C_α_ = C_β_ symmetric stretching peak toward higher wavenumbers. Thus, deconvolution of this main signal can provide a quantitative estimation of the oxidation level of PEDOT. Indeed, it was reported that the integrated areal ratio of these two signals, resulting from the neutral and oxidized structures in PEDOT, is proportional to the doping level of the conductive polymer.^54,55^ Following this approach, a value of ∼0.2 was obtained for NAF, NAF-D, and NAF-E, which is in accordance with the doping level of a commercially available PEDOT/PSS (PH-1000) calculated in the literature with the same method.^56^ This confirms that a comparable amount of charge carriers is present in NAF, NAF-D, and NAF-E, thereby suggesting that the larger capacitance of NAF-D and NAF-E is probably ascribed to a more efficient diffusion of the electrolyte inside the conductive film, if compared to NAF, as a result of a structural and interfacial rearrangement of the films in the presence of these polar organic solvents.

### Surface Analysis

**Figure 5:**
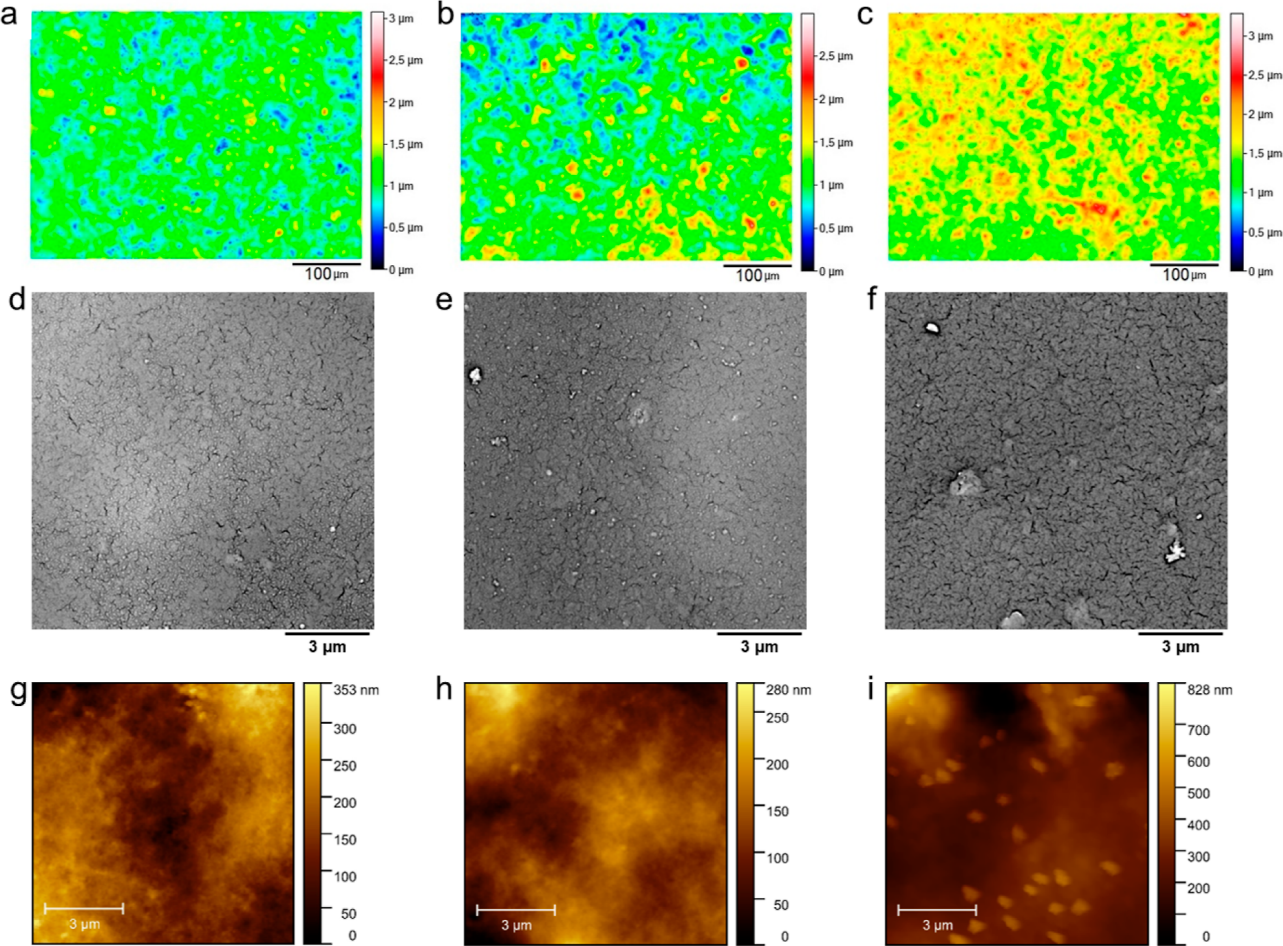
Surface profile of (a) NAF, (b) NAF-D, and (c) NAF-E films determined based on optical profilometry; SEM images of (d) NAF, (e) NAF-D, and (f) NAF-E films; and AFM images of (g) NAF, (h) NAF-D, and (i) NAF-E films.

Coating of FTO electrodes with NAF films produced morphologically homogeneous and compact films, as evidenced from their surface profiles and SEM and AFM images (Figure 5). In all NAFs films, but particularly in the case of NAF-E, the formation of interconnected micrograins could be observed (Figure 5d–f). These grains are spherical in shape and separated by a small distance with respect to the grain size, and still they are interlinked to each other. Previous studies on PEDOT/PSS materials treated with DMSO concluded that these highly clustered grains are expected to play a key role in charge transfer mechanism in the system.^48^ Additionally, AFM images of NAF-E (Figure 5i) clearly showed the formation of polymer agglomerates when the excess of PSS is removed by the addition of EG, as observed previously.^57^

Interestingly, thickness analysis (Table 1) revealed a contraction of the conductive film when NAF was treated with DMSO or EG. In particular, the thickness of pristine NAF films is on the order of 2.0 μm, whereas for NAF-D and NAF-E, it is approximately 1.6 μm. The reduction of the film thickness is consistent with a reduction of its overall volume since coatings were fabricated by keeping constant both the geometrical area and the volume of the drop-casting solution. Considering that the overall amount of charge carriers is not affected by the treatment of NAF with either DMSO or EG, a reduction of the film volume reflects a larger charge carrier density. As already noted for PEDOT/PSS, treatment with DMSO has been found to induce a distinct phase separation within the polymer by facilitating the aggregation of the PEDOT macromolecules allowing for the formation of an electrically percolated network with improved conductivity.^58^ AFM analysis (Table 1) revealed that it is NAF-E that exhibits the highest roughness in the nanoscale (76.5 ± 6.5 nm), which comes from the presence of polymer agglomerates.^57^ Although it could be expected that higher nanoroughness should result in higher electroactive area, in the case of NAF-E, the increase in roughness is derived from the presence of loosely packed clusters, i.e., phase-separated PEDOT islands. To sum up, the morphological investigation confirms that, within the explored series, the largest capacitance observed for NAF-D is due to a more effective activation of innermost charge carriers in PEDOT (Figure S1b).

**Table 1:** Values of Thickness and Roughness of NAF, NAF-D, and NAF-E Films

	NAF	NAF-D	NAF-E
**thickness, μm**	2.0 ± 0.1	1.6 ± 0.1	1.6 ± 0.1
**roughness, nm**	49.2 ± 2.5	39.0 ± 3.6	76.5 ± 6.5

### Charge–Discharge Characteristics

**Figure 6:**
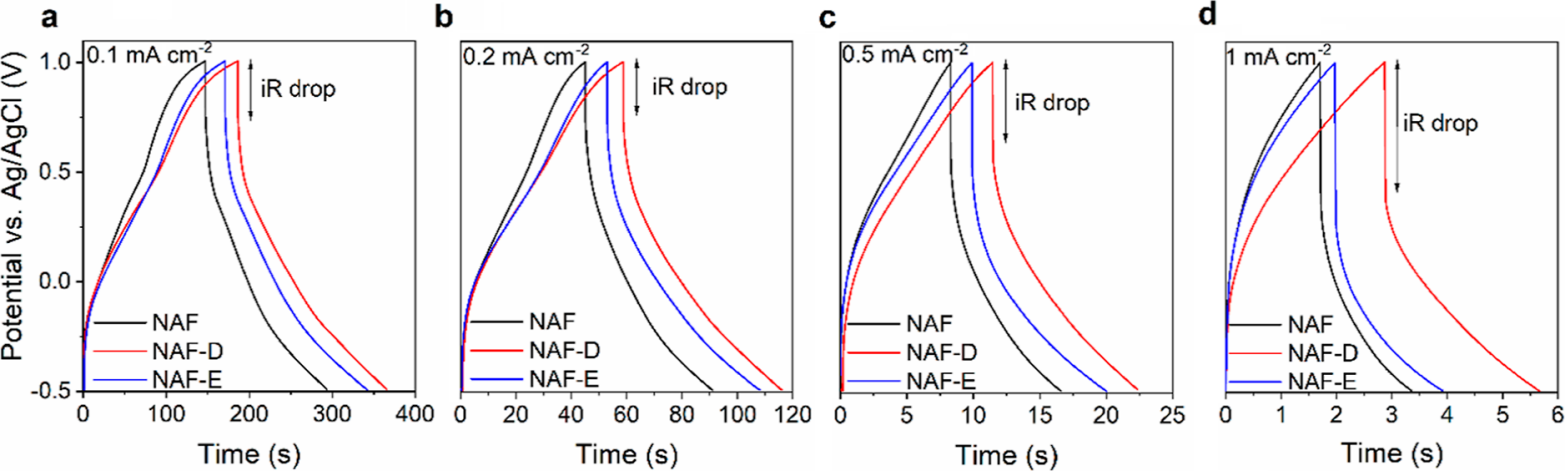
Galvanostatic charging–discharging curves for NAFs collected in 1 M Na_2_SO_4_ solution at (a) 0.1 mA cm^–2^; (b) 0.2 mA cm^–2^; (c) 0.5 mA cm^–2^, and (d) 1 mA cm^–2^.

**Figure 7:**
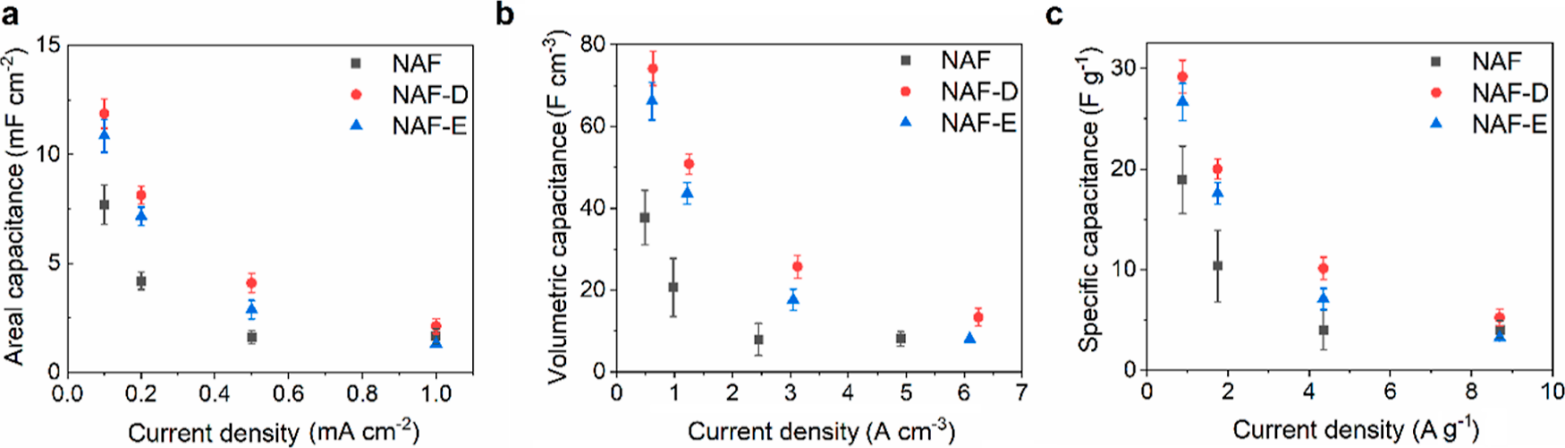
Comparison of capacitive properties of NAFs: (a) areal capacitance vs current density; (b) volumetric capacitance vs current density; and (c) specific capacitance vs current density.

Galvanostatic charge–discharge (GCD) curves (Figure 6a–d) were collected using a 1 M Na_2_SO_4_ solution to investigate capacitance, Coulombic efficiency, energy, and power of NAF, NAF-D, and NAF-E films (Figure 7a–c). Resulting GCD curves exhibit a linear nature without the presence of any plateau regions, demonstrating the electrostatic charge storage mechanism of NAFs at the current densities of 0.1, 0.2, 0.5, as well as 1 mA cm^–2^. Small *iR* losses observed as vertical regions of GCD curves (Figures 6a–d, S4) indicate that all NAFs exhibit low uncompensated resistance, particularly at low current densities of charging, which avoids undesirable voltage changes across electrochemical interfaces. Both NAF-D and NAF-E are also characterized by longer charging and discharging times than those of NAF, particularly at higher current densities.

It is well-known that capacitance of PEDOT scales with film volume,^59^ though the volumetric capacitance becomes saturated at larger volumes due to ion diffusion limitations.^40^ Such restriction is, however, not present for areal capacitance values. According to the GCD curves and knowing the thickness of NAFs, as well as the mass of the material, it was possible to calculate areal capacitance, volumetric capacitance, and specific capacitance (Figure 7a–c). In all cases, the highest values of capacitance are noted for NAF-D, which are only slightly higher than NAF-E, while both outperform NAF. The highest areal capacitance (11.9 ± 0.7 mF cm^–2^), volumetric capacitance (74.2 ± 4.2 F cm^–3^), and specific capacitance (29.2 ± 1.6 F g^–1^) values are noted for NAF-D at the current density of 0.1 mA cm^–2^ (corresponding to 0.6 A cm^–3^ and 0.9 A g^–1^). Interestingly, the volumetric capacitance of PEDOT/PSS has been estimated as 39 F cm^–3^,^59^ i.e., two times lower than that noted for NAF-D. This increase in volumetric capacitance is expected to result from the increase in the density of sites, occurring due to the presence of Nafion and a secondary dopant.^19,59^

**Figure 8:**
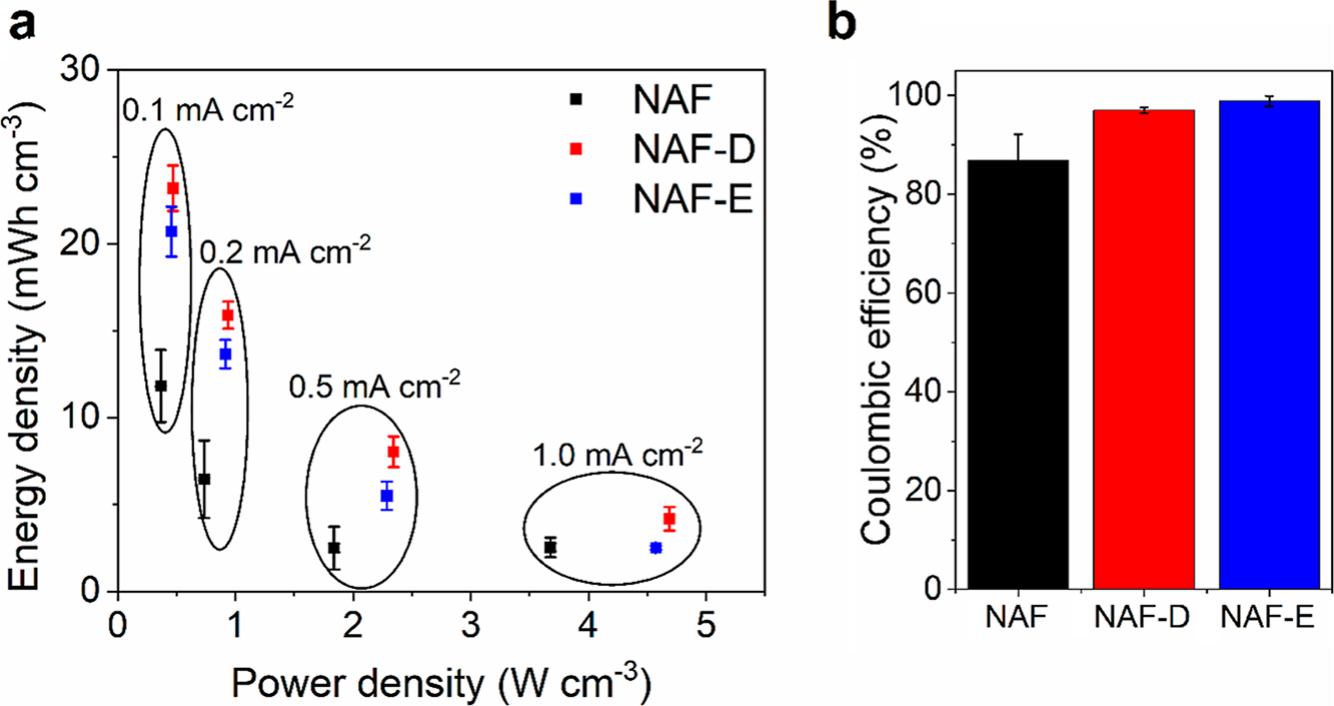
Comparison of energy storage performance of NAFs: (a) Ragone plot; (b) Coulombic efficiency.

Consequently, both NAF-D and NAF-E show excellent performance as supercapacitors (Figure 8a and Table S2), with NAF-D reaching an energy density of 23.1 ± 1.5 mWh cm^–3^ at the power density of 0.47 ± 0.01 W cm^–3^, particularly when compared with other PEDOT/PSS based supercapacitive materials reported in the literature, e.g., MnO_2_–PEDOT/PSS (0.38 W cm^–3^ and 0.362 mWh cm^–3^),^60^ PEDOT/PSS–poly(acrylonitrile) nanofiber composite (0.83 W cm^–3^ and 9.56 mWh cm^–3^),^61^ as well as PEDOT/PSS-polyaniline (0.98 W cm^–3^ and 11.9 mWh cm^–3^).^62^ It should be noted that the increase in supercapacitive performance is realized purely by the use of Nafion as a primary dopant, without the need to add any electroactive fillers, such as MnO_2_ or polyaniline. Besides, the presence of both DMSO and EG is found to increase the Coulombic efficiency from 87 ± 5% (NAF) to 97 ± 1 and 99 ± 1% (NAF-D and NAF-E, respectively) (Figure 8b), indicating excellent charge and discharge reversibility of the supercapacitors.

The comparison of the surface morphology of NAF-based films before and after electrochemical analysis (Figures S5,S6) revealed that electrochemical tests affected the integrity of the NAF coating. Particularly in the case of NAF-D, large cracks were uniformly formed over the whole area of the coating (Figure S5). AFM images (Figure S6) clearly showed further clustering of PEDOT grains to form larger agglomerates, partially affecting the continuity of an electrically percolated network. In all cases, electrochemical treatment resulted in an increase in surface roughness, and the largest change was noted for NAF-E.

### Supercapacitor Design

**Figure 9:**
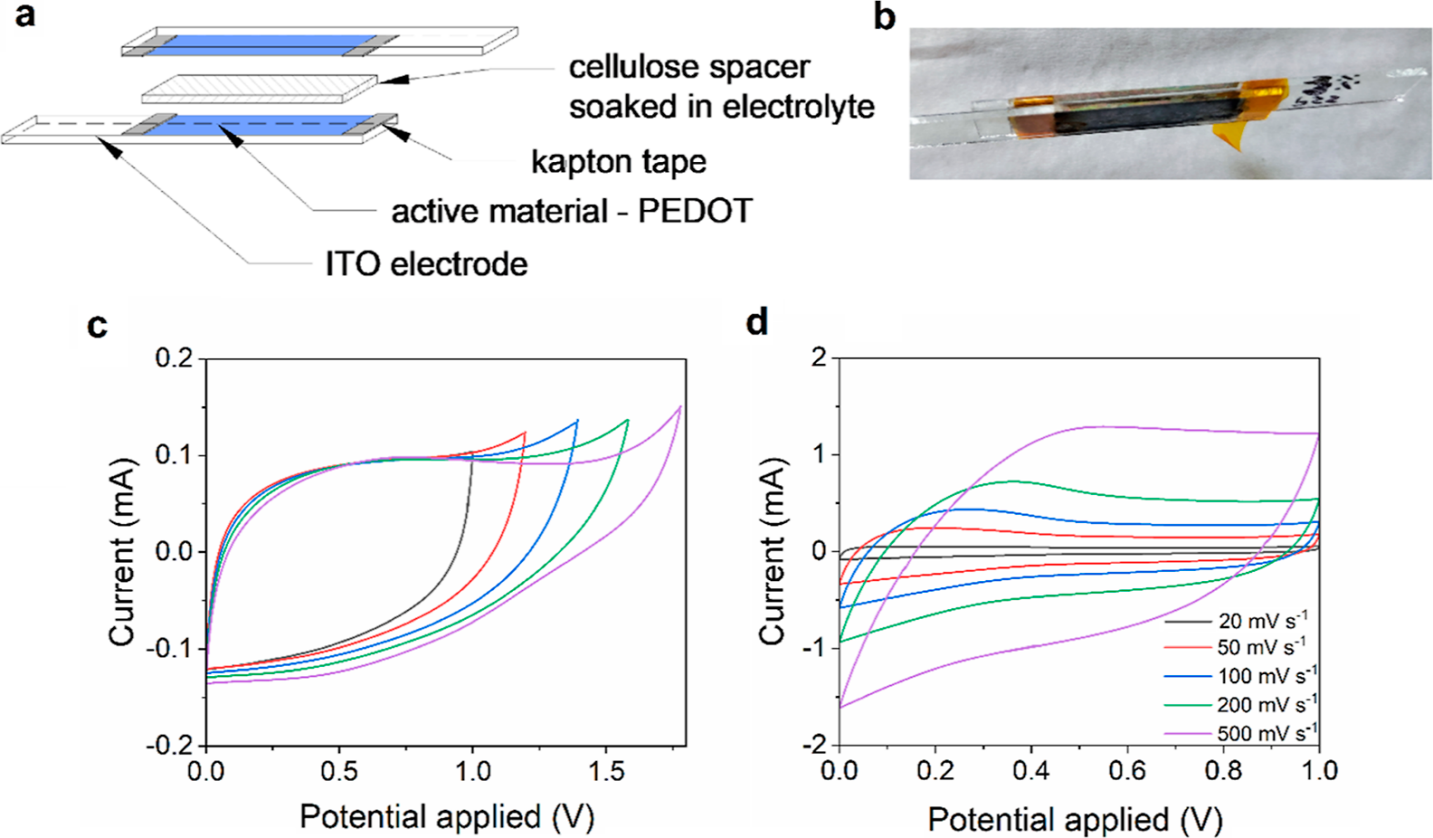
Electrochemical analysis of NAF-D-based SC device: (a) schematic diagram of the device and (b) digital photograph of the device; cyclic voltammetry curves collected in 0.2 M Bu_4_NPF_6_ in CH_3_CN/PC (1:3) in (c) different potential rages and (d) different scan rates.

To validate the performance of PEDOT/Nafion as a supercapacitive material, symmetric supercapacitors (SCs) were fabricated by depositing the same material (either PEDOT/PSS or NAF-D) on each electrode (Figure 9a–b). In particular, devices fabricated with NAF-D were compared with devices based on PEDOT/PSS, which was used as a reference material owing to its well-known supercapacitive behavior. An organic electrolyte based on propylene carbonate/acetonitrile containing 0.2 M Bu_4_NPF_6_ (TBAH) was used here due to its extended electrochemical stability window compared to water and its better suitability to long-term stability testing of the devices.

Preliminary CV tests showed that NAF-D remains stable even when a large potential of 1.75 V is applied (Figure 9c). Considering that ITO was chosen as the substrate, further tests were conducted in the potential range between 0 and 1 V to avoid ITO damage. The scan rate was varied in the range between 20 and 500 mV s^–1^. A nearly rectangular shape of the CVs was obtained at low scan rates, indicating almost ideal capacitive behavior. However, at higher scan rates, the resulting CVs were not ideally rectangular, which reveals contribution of electrochemical pseudocapacitance (Figure 9d). To further study the pseudocapacitive contribution to electrochemical energy storage in NAF-based SC, the power law (eq 8) and the plot ln (*i*) versus ln (*v*) (Figure S7) were used. For all investigated potentials (0.4, 0.6, and 0.8 V), the slope of a ln (*i*) versus ln (*v*) plot (parameter b) was close to 1, indicating dominant pseudocapacitance contribution in NAF-based SC.^37^ Since we operated with relatively thick layers (1.6–2.0 μm), we expect that it is a mix of intercalation redox pseudocapacitance.

**Figure 10:**
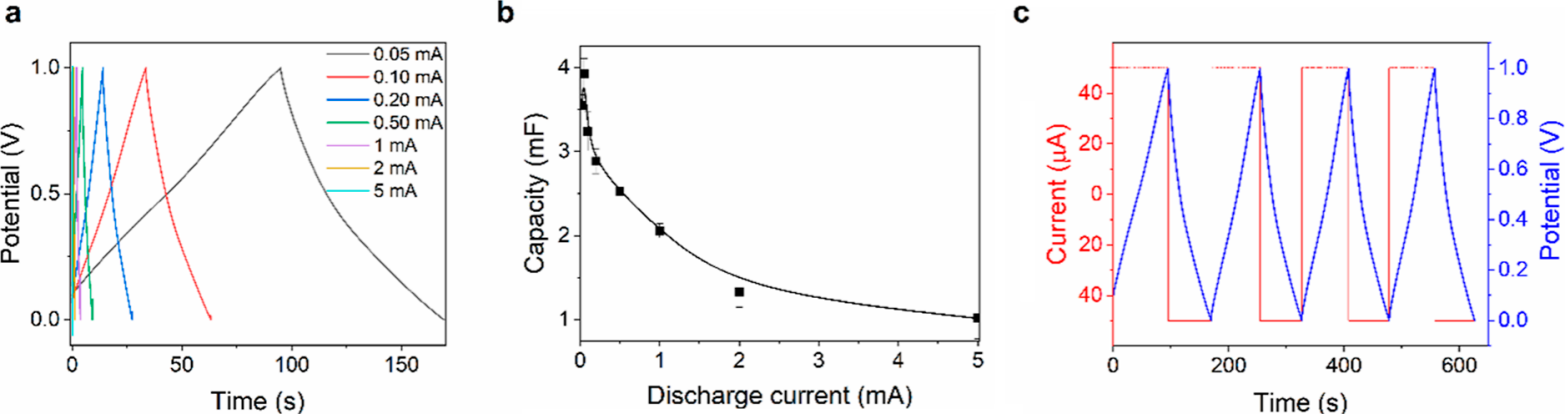
Galvanostatic charging–discharging behavior of a SC device based on NAF-D: (a) galvanostatic charging–discharging curves for NAF-D in the current range 50–5000 μA and the voltage limits between 0 and 1 V; (b) capacity vs discharge current; and (c) current and potential vs time.

**Figure 11:**
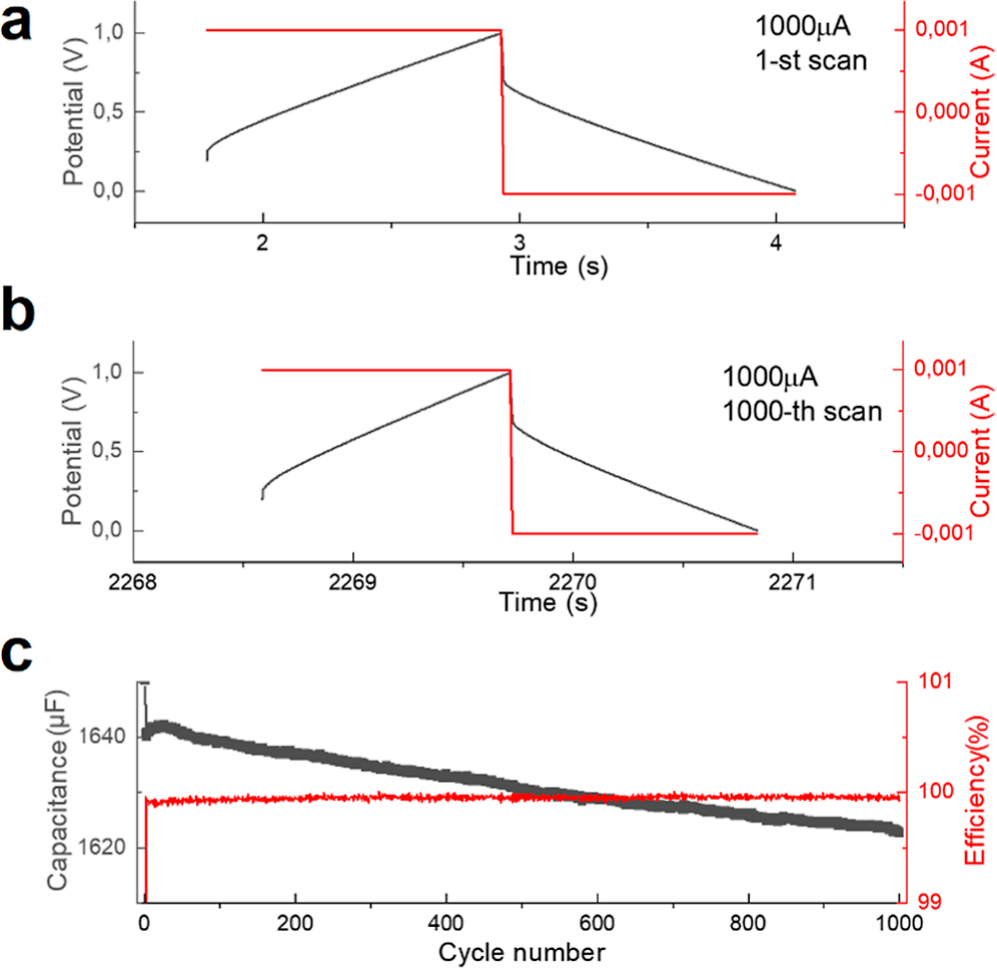
Electrochemical stability of SC devices based on NAF-D evaluated through multiple charging/discharging: (a) first cycle and (b) 1000th cycle of a charging/discharging process of a SC; (c) changes of capacitance (registered during discharging) and Coulombic efficiency (charge required for discharging vs charging) with the cycle number. Current of 1 mA was applied for both charging and discharging processes.

SC devices were tested by repetitive constant current charging/discharging, applying current in the range of 50–5000 μA and the voltage limits between 0 and 1 V (Figure 10). The curves exhibit a typical triangular shape indicating good capacitive characteristics. However, the presence of *iR* drop is noticeable, especially at higher current densities, and is ascribed to the series resistance of the device, incorporating the sum of the ohmic, charge transfer and diffusional contributions. Particularly, the latter is expected to be relatively high in a viscous organic solvent in the presence of large cations like TBA^+^. The resulting capacitance values varied with applied current, reaching maximum capacitance of approximately 3.9 ± 0.2 mF for the discharge current of 50 μA (Figure 10c), with a specific capacitance of approximately 15.7 F g^–1^ (related to the mass of the active material on both electrodes) or 1.8 mF cm^–2^ (related to the cross-section surface). Reaching maximum capacity requires approximately 79 s, while discharging takes 70 s (Coulombic efficiency η = 88.6%). Charging with a higher current shortens the charging and discharging to fractions of a second and increases efficiency but also results in halving the capacity. For comparison, twin devices fabricated with commercial PEDOT–PSS yielded, under similar conditions, a capacitance of 2.7 ± 0.1 μF (≈10.6 F g^–1^ or 1.2 μF cm^–2^). It is worth noting that all capacitors exhibited high stability, as confirmed by the maintenance of their initial characteristics (e.g., capacitance) even after repeated charging/discharging processes (Figure 11). During 1000 cycles of charging/discharging, the efficiency remains above 99% and the capacitance drops by only approximately 1.3%.

## Conclusions

In this paper, PEDOT/Nafion, and particularly solvent-treated PEDOT/Nafion films, were proposed as promising materials for the design of supercapacitors. The electrochemical investigations of NAFs revealed their high areal capacitance (reaching 22.2 ± 0.8 mF cm^–2^ at 10 mV s^–1^ for NAF-D) and low charge transfer resistance (379 ± 19 Ω for NAF-E), being the result of a more effective ion diffusion inside the conductive film, as confirmed by the results of the spectroscopic studies. A reduction in film volume, as prompted by secondary doping, together with the unchanged overall amount of charge carriers clearly states that solvent treatment is effective to increase the charge carrier density. As a result, NAF-D exhibits an excellent volumetric capacitance of 74.2 ± 4.2 F cm^–3^, with a Coulombic efficiency of 99% and an energy density of 23.1 ± 1.5 mWh cm^–3^ at a power density of 0.5 W cm^–3^. A proof-of-concept of symmetric supercapacitor based on PEDOT/Nafion exhibits a specific capacitance of approximately 15.7 F g^–1^ and impressive long-term stability, indicating PEDOT/Nafion as a promising material for energy storage applications. Taking into consideration well-known cation exchange properties of Nafion, exhibiting superselectivity and facile cation transport,^27^ it is expected that PEDOT/Nafion could also be used as a carrier of small cations, e.g., lithium, going toward the realization of a novel concept of hybrid supercapacitors containing elements of a lithium-ion cell together with a supercapacitor.^63,64^

## Supporting Information Available

Cyclic voltammetric curves in the presence of a redox probe; summary of the electrical properties of NAFs; ATR-FTIR spectra of NAF, NAF-D, and NAF-E; chemical structure of PEDOT with marked C_α_, C_β_, and C_α’_ atoms; *iR* drop values for NAFs; summary of the energy storage performance of NAFs; SEM images of NAF, NAF-D, and NAF-E before and after electrochemical analysis; AFM images and roughness of NAF, NAF-D, and NAF-E before and after electrochemical analysis; and power law dependence of the peak current on sweep rate for NAF-D based SC device (PDF)
