# Supplementary material for: PEDOT:Nafion for Highly Efficient Supercapacitors
Source: ACS Appl Mater Interfaces. 2024 Apr 23;16(18):23253–64. doi: 10.1021/acsami.4c01085 (PMC11082849; doi:10.1021/acsami.4c01085)
Supplement: Supplementary file 1 — am4c01085_si_001.pdf [file am4c01085_si_001.pdf]

Supporting Information

PEDOT:Nafion for highly efficient supercapacitors

Małgorzata Skorupa[1,2], Krzysztof Karoń[1,3], Edoardo Marchini,[4] Stefano Caramori,[4] Sandra Pluczyk-Małek,[1,3] Katarzyna Krukiewicz[1,3], Stefano Carli[5*]*

[1]Department of Physical Chemistry and Technology of Polymers, Silesian University of Technology, M. Strzody 9, 44-100 Gliwice, Poland

[2]Joint Doctoral School, Silesian University of Technology, Akademicka 2A, 44-100 Gliwice, Poland

[3]Centre for Organic and Nanohybrid Electronics, Silesian University of Technology, S. Konarskiego 22B, 44-100 Gliwice, Poland

[4]Department of Chemical, Pharmaceutical and Agricultural Sciences, University of Ferrara, 44121 Ferrara, Italy

[5]Department of Environmental and Prevention Sciences, University of Ferrara, 44121 Ferrara, Italy

* Corresponding authors: Stefano Carli (crlsfn@unife.it)
Katarzyna Krukiewicz (katarzyna.krukiewicz@polsl.pl)

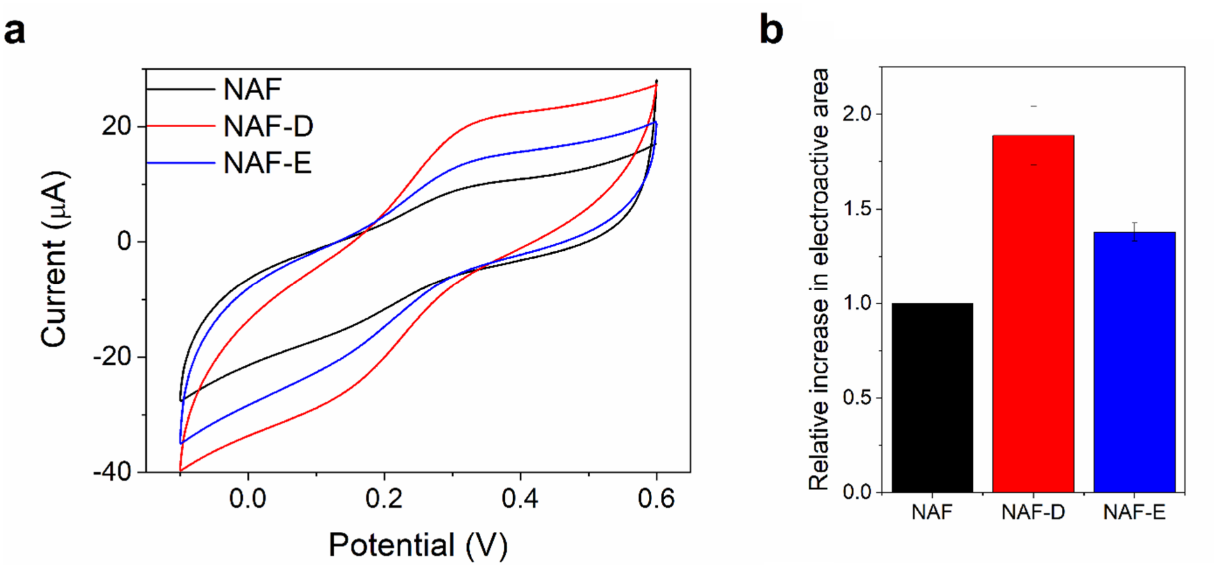

Figure S1. Electrochemical analysis of NAFs: a) cyclic voltammetric curves collected in 0.1 M KCl (aq) in the presence of a redox probe, 5 mM $K_3[Fe(CN)_6]$, at the scan rate of 5 mV/s; b) relative increase in electroactive surface area.

Table S1. Summary of the electrical properties of NAFs derived from the equivalent circuit analysis.

	**NAF**	**NAF-D**	**NAF-E**
Solution resistance: R_S [Ω]	105 ± 2	116 ± 4	98 ± 3
Charge transfer resistance: R_{CT} [Ω]	1 229 ± 36	751 ± 44	379 ± 19
Warburg diffusion: A_w	330 ± 39	597 ± 70	445 ± 35
Constant phase element: $P \cdot 10^5$	4.862 ± 0.121	1.921 ± 0.108	1.516 ± 0.103
Constant phase element: n	0.887 ± 0.006	0.915 ± 0.011	0.935 ± 0.010
Characteristic frequency, Hz	15	45	90
Goodness of fit: χ^2	0.00173	0.00668	0.00455

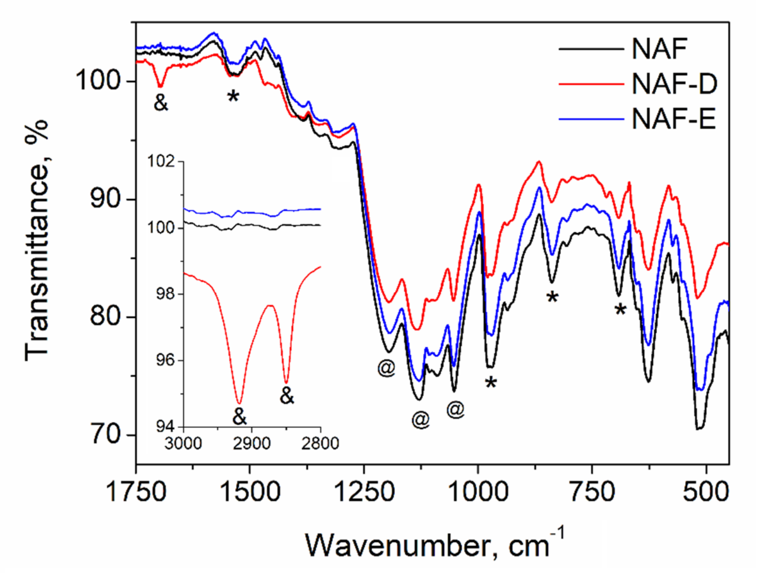

Figure S2. ATR-FTIR spectra of NAF, NAF-D and NAF-E, with the characteristic peaks marked for PEDOT (*), Nafion (@), and DMSO (&).

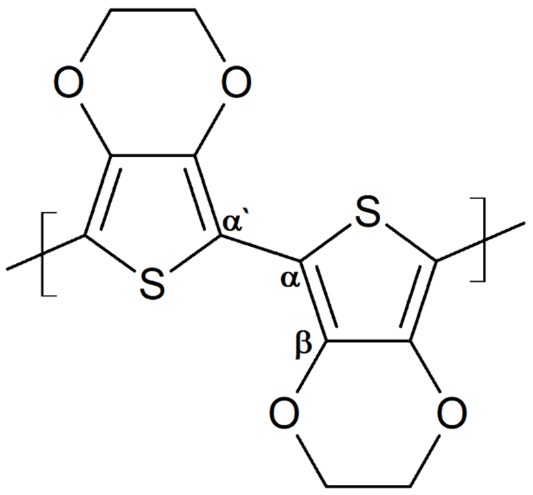

Figure S3. Chemical structure of PEDOT with marked C_α, C_β and $C_{\alpha'}$ atoms.

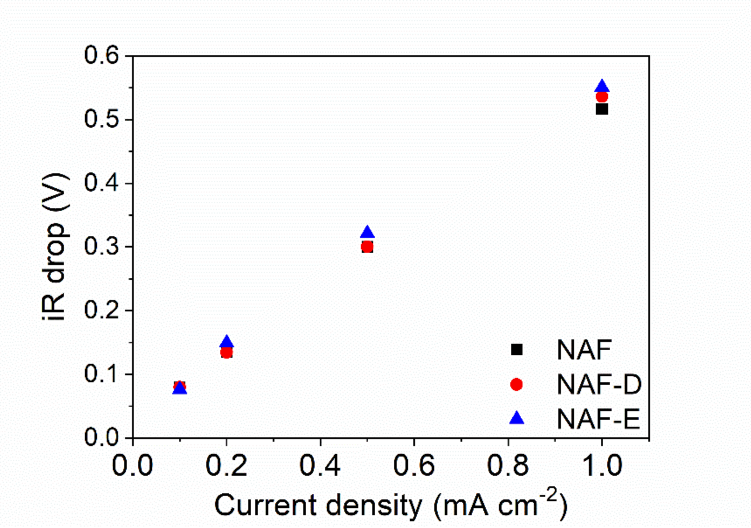

Figure S4. iR drop values for NAF, NAF-D and NAF-E films with respect to the current density of charging and discharging processes.

Table S2. Summary of the energy storage performance of NAFs; I – current density, $C_{GCD(A)}$ – areal capacitance, $C_{GCD(V)}$ – volumetric capacitance, $C_{GCD(S)}$ – specific capacitance, P – power density, E – energy density.

	**I** **[mA cm^{-2}]**	**$C_{GCD(A)}$** **[mF cm^{-2}]**	**$C_{GCD(V)}$** **[F cm^{-3}]**	**$C_{GCD(S)}$** **[F g^{-1}]**	**P** **[W cm^{-3}]**	**E** **[mWh cm^{-3}]**
**NAF**	0.1	7.7 ± 0.9	37.8 ± 6.7	18.9 ± 3.3	0.37 ± 0.06	11.8 ± 2.1
**NAF-D**		11.9 ± 0.7	74.2 ± 4.2	29.2 ± 1.6	0.47 ± 0.01	23.1 ± 1.5
**NAF-E**		10.8 ± 0.8	66.2 ± 4.6	26.7 ± 1.8	0.46 ± 0.03	20.7 ± 1.4
**NAF**	0.2	4.2 ± 0.4	20.6 ± 7.1	10.3 ± 3.6	0.74 ± 0.25	6.4 ± 2.2
**NAF-D**		8.1 ± 0.4	50.8 ± 2.5	20.0 ± 1.0	0.94 ± 0.04	15.9 ± 0.8
**NAF-E**		7.2 ± 0.4	43.6 ± 2.6	17.6 ± 1.1	0.91 ± 0.06	13.6 ± 0.8
**NAF**	0.5	1.6 ± 0.3	8.0 ± 3.9	4.0 ± 2.0	1.83 ± 0.91	2.5 ± 1.2
**NAF-D**		4.1 ± 0.5	25.7 ± 2.8	10.1 ± 1.1	2.34 ± 0.26	8.0 ± 0.9
**NAF-E**		2.9 ± 0.4	17.6 ± 2.6	7.1 ± 1.1	2.29 ± 0.34	5.5 ± 0.8
**NAF**	1.0	1.6 ± 0.4	8.1 ± 1.8	8.7 ± 4.0	3.68 ± 0.81	2.5 ± 0.6
**NAF-D**		2.1 ± 0.3	13.3 ± 2.2	5.2 ± 0.8	4.67 ± 0.76	4.2 ± 0.7
**NAF-E**		1.3 ± 0.1	8.0 ± 0.4	3.2 ± 0.2	4.57 ± 0.22	2.5 ± 0.1

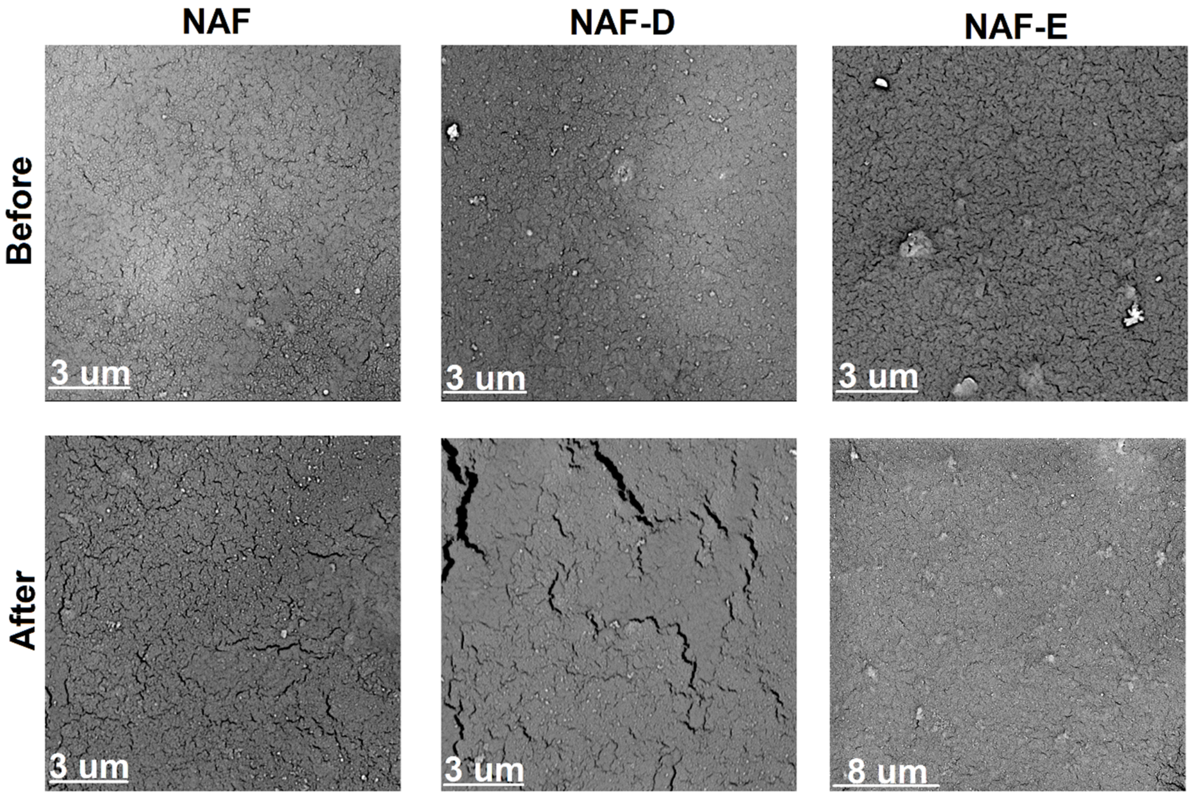

Figure S5. SEM images of NAF, NAF-D and NAF-E before and after electrochemical analysis.

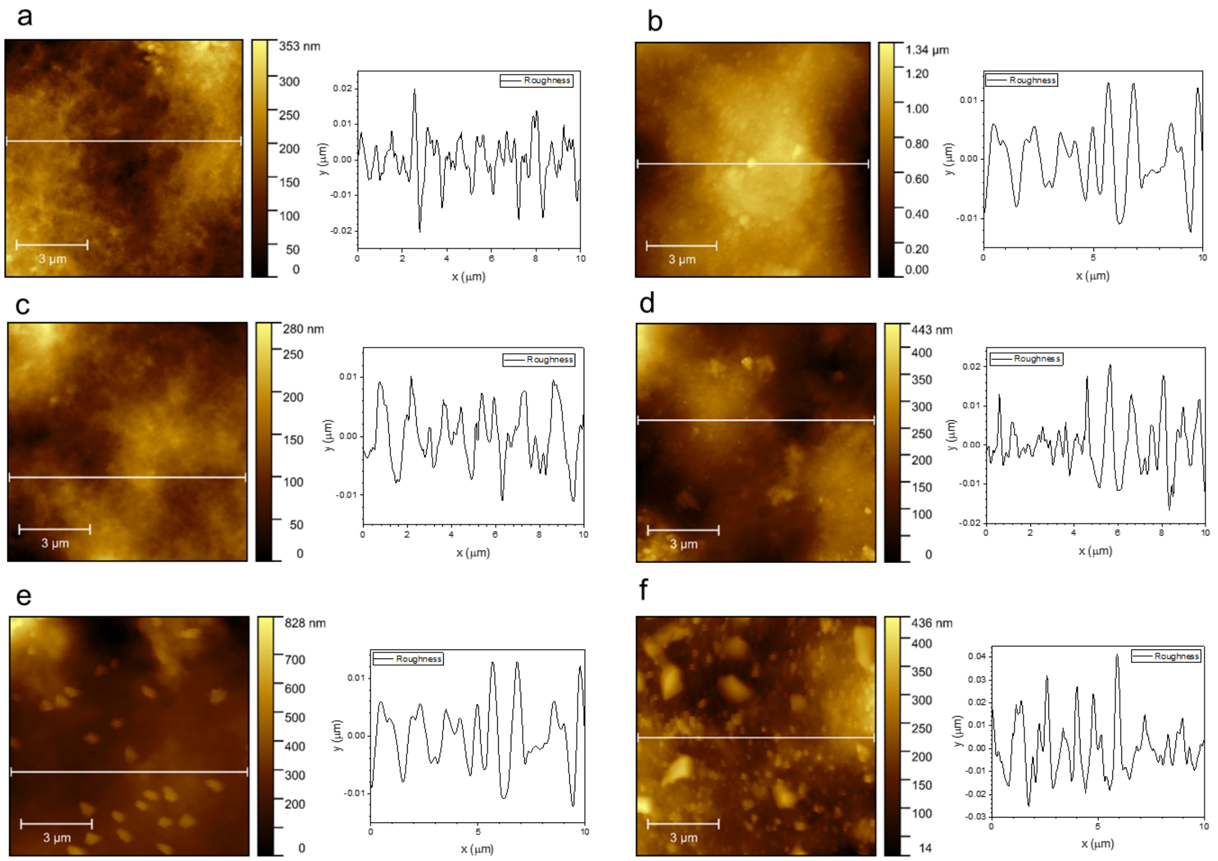

Figure S6. AFM images and roughness of NAF (a,b), NAF-D (c,d) and NAF-E (e,f) before (a,c,e) and after (b,d,f) electrochemical analysis.

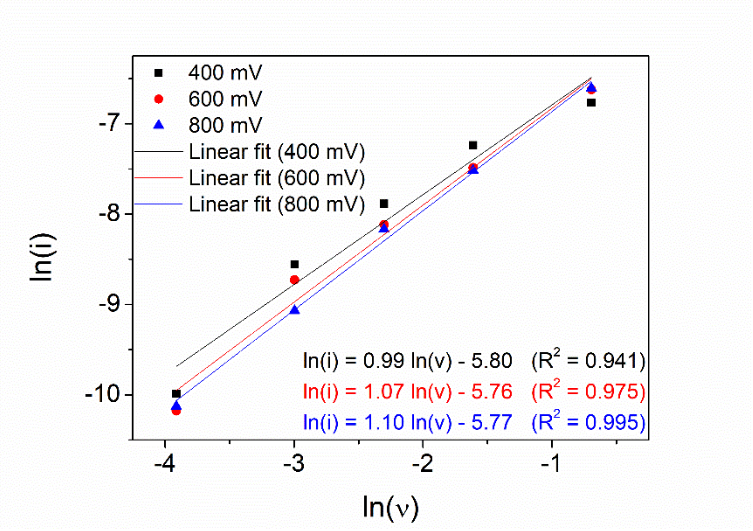

Figure S7. Power law dependence of the peak current on sweep rate for NAF-D based SC device.
